# Cell dedifferentiation and epithelial to mesenchymal transitions during intestinal regeneration in *H. glaberrima*

**DOI:** 10.1186/1471-213X-11-61

**Published:** 2011-10-17

**Authors:** José E García-Arrarás, Griselle Valentín-Tirado, Jaime E Flores, Rey J Rosa, Angélica Rivera-Cruz, José E San Miguel-Ruiz, Karen Tossas

**Affiliations:** 1 Biology Department, University of Puerto Rico, Rio Piedras 00931, Puerto Rico

## Abstract

**Background:**

Determining the type and source of cells involved in regenerative processes has been one of the most important goals of researchers in the field of regeneration biology. We have previously used several cellular markers to characterize the cells involved in the regeneration of the intestine in the sea cucumber *Holothuria glaberrima*.

**Results:**

We have now obtained a monoclonal antibody that labels the mesothelium; the outer layer of the gut wall composed of peritoneocytes and myocytes. Using this antibody we studied the role of this tissue layer in the early stages of intestinal regeneration. We have now shown that the mesothelial cells of the mesentery, specifically the muscle component, undergo dedifferentiation from very early on in the regeneration process. Cell proliferation, on the other hand, increases much later, and mainly takes place in the mesothelium or coelomic epithelium of the regenerating intestinal rudiment. Moreover, we have found that the formation of the intestinal rudiment involves a novel regenerative mechanism where epithelial cells ingress into the connective tissue and acquire mesenchymal phenotypes.

**Conclusions:**

Our results strongly suggest that the dedifferentiating mesothelium provides the initial source of cells for the formation of the intestinal rudiment. At later stages, cell proliferation supplies additional cells necessary for the increase in size of the regenerate. Our data also shows that the mechanism of epithelial to mesenchymal transition provides many of the connective tissue cells found in the regenerating intestine. These results present some new and important information as to the cellular basis of organ regeneration and in particular to the process of regeneration of visceral organs.

## Background

In recent years, investigators have shown a renewed interest in regenerative phenomena. In view that many "classical" model system organisms show limited regenerative capacities, research on non-traditional model systems has flourished. Many of these organisms, such as planarians and *Hydra*, had been studied previously, some for almost three centuries. However, modern cellular and molecular tools have permitted a novel look into these regeneration models and a re-examination of the cellular and molecular mechanisms involved in the regenerative events [1].

Crucial to the understanding of organ or limb regeneration is identifying the origin of the cells that form the new regenerated structure. Equally important is a related issue, whether the cells undergo dedifferentiation and/or proliferation. Experimental results have shown some similarities and differences among regenerating animal groups. For example, in planarians, regeneration depends on a population of proliferating stem cells, called neoblasts, that can give rise to all cell phenotypes [2,3]. In contrast, during newt limb regeneration, cells adjacent to the injury dedifferentiate, proliferate and then give rise to the cells of the regenerating structure [4,5].

Our laboratory has been active in studying the process of intestinal regeneration using the sea cucumber *Holothuria glaberrima *as a model system. This species, like many other holothurians, has the capacity to eject its digestive tract under stressful environmental circumstances [6]. The process occurs naturally and can be induced in the laboratory [7]. Following the evisceration process, the animal regenerates the lost digestive tract, which is mainly composed of descending and ascending small intestine and a large intestine. The new intestine is formed at the edge or margin of the mesentery where the eviscerated intestine was previously attached. We have shown that the new intestine forms from a thickening of the mesentery [7]. This thickening forms a solid rod that extends from the cloaca to the esophagus. As regeneration proceeds, cells from the lumen of the esophagus and the cloaca migrate into the intestinal rudiment forming its lumen. Roughly a month after regeneration has begun, a smaller but apparently functional new intestine has formed.

We have investigated the cellular and molecular events that occur during this regenerative organogenesis. Our initial studies showed that intestinal regeneration involved cell division [7], cell migration [8], extracellular matrix remodeling [9] and cell dedifferentiation [10]. Many of these mechanisms are common to regenerative events not only in other echinoderms [11-13], but in most animals with strong regenerative capacities, such as Hydra [14-16], Planaria [2,3], and some amphibians [17]. More recently we have probed the molecular basis of intestinal regeneration. Using gene-by-gene strategies, high throughput sequencing and microarrays we have now identified multiple genes that are associated with the process of intestinal regeneration [18-21].

In our quest for cellular markers that identify cell populations or phenotypes associated with the intestinal regenerative phenomenon, we obtained a monoclonal antibody that labels the intestinal mesothelium. This is a composite tissue in echinoderms, made up of peritoneocytes (or coelomic epithelial cells) and myocytes [22]. Mesothelial cells are known to play a key role in intestinal regeneration [12]. This antibody has now been used to probe the spatial and temporal pattern of previously described cellular events that occur during the first ten days of intestinal regeneration, namely muscle dedifferentiation and cell proliferation. More importantly, by studying the expression pattern of the cells recognized by this novel antibody during the regenerative event we made the surprising discovery that the cells of the mesothelium are ingressing into the underlying connective tissue to give rise to mesenchymal cells within the regenerating structure. This novel phenomenon has not been described in other regenerating echinoderms. Finally, we have integrated the available information into a coherent view of the cellular origins that lead to the formation of the intestinal rudiment.

## Results

### Overview of intestinal regeneration

**Figure 1:**
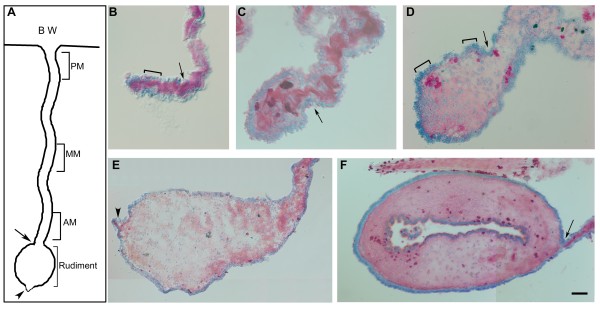
**Stages of intestinal regeneration in *H. glaberrima***. (A) Diagram showing the relationship of the intestinal rudiment (brackets) to the mesentery and body wall (BW). The mesentery is divided into three sections: Proximal to the body wall (PM), medial mesentery (MM) and adjacent to the rudiment (AM). Transverse tissue sections of mesentery and regenerating intestine at (B) 1-, (C) 3-, (D) 5-, (E) 7- and (F) 10-days post evisceration (dpe) were stained with Toluidene Blue. (B) At 1-dpe the coelomic epithelium covers the tip of the mesentery but there is no clear thickening. (C) By 3-dpe, a small enlargement can be observed at the mesenterial tip. (D) By 5-dpe the intestinal rudiment has increased considerably in size and some areas of the mesothelium appear to have an increased number of cells (brackets) when compared to the 1-day mesentery (see brackets in Figure 1B). (E) By 7-dpe the rudiment has acquired a pear-shaped morphology and the appendix at the tip (arrowhead) is evident (F) At 10-dpe the lumen has formed and all tissue layers of the mature intestine can be found within the rudiment. Arrows signal the boundary between the forming intestinal rudiment and the mesentery. Bar = B-C 25 μm, D 50 μm, E&F 100 μm

**Figure 2:**
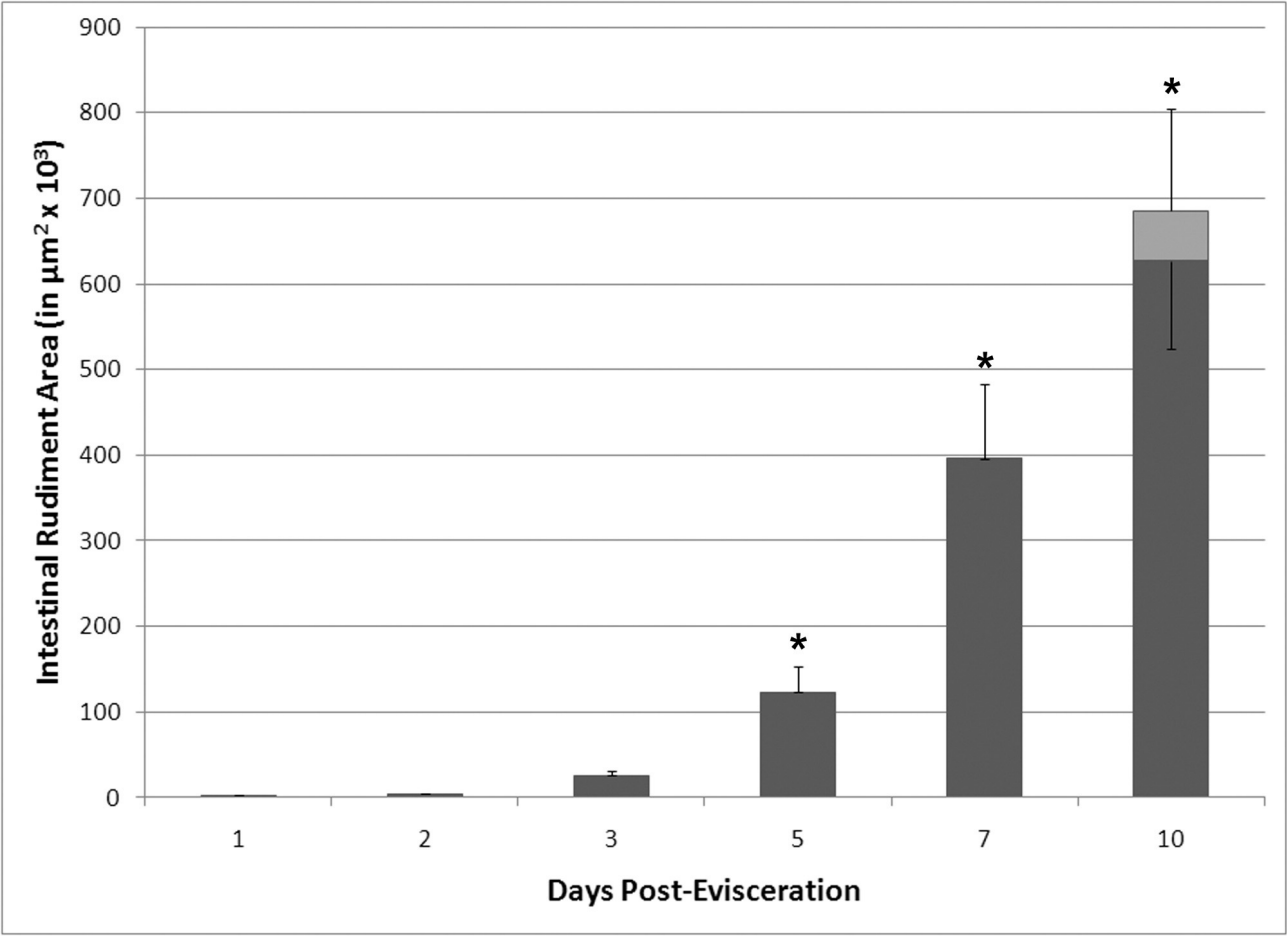
**Quantification of the area of the intestinal rudiment during the process of intestinal regeneration**. The area encompassed by the thickening of the mesentery was measured in transverse sections. The main growth of the structure begins 3 days after eviscerations. At 10 days a lumen has formed in all animals, thus the black bar at 10 days denotes only the area of tissue (does not include the lumen area), while the gray bar represents the total area of the intestine (including the area encompassed by the lumen). Each point represents the mean percentage ± S.E. of at least 3 animals. *p < 0.05.

To understand the results described here, it is necessary to provide background information on the tissue and morphological changes that underscore the regenerative process. Some of these events have been described in previous publications, however, they have never been presented in a cohesive view that shows the sequence of events of early regeneration stages. Tissue labeling with Toluidene Blue provides the tissue/organ level information needed (Figure 1). The quantification of the growth is shown in Figure 2. We have also provided a drawing (Figure 1A) depicting the relationship of the growing rudiment (~5-dpe) to the mesentery and that of the mesentery to the body wall. This figure provides a point of reference for the findings described below.

As has been shown before, the formation of the intestinal rudiment takes place at the free end of the intestinal mesentery. One day after evisceration, epithelial cells covered the cut edge of the mesentery. However, no thickening or any other obvious morphological difference could be observed between the tip and the rest of the remaining mesentery (Figure 1B). The first indication of swelling of the distal mesentery was observed in some animals at day 2 and in all animals by day 3 of regeneration (Figure 1C). The thickening was wider near the edge of the mesentery and became thinner as one moved toward the body wall gradually achieving the width of the rest of the mesentery. This growth of the distal portion of the mesentery was mainly due to an accumulation of cells at the free edge, where the mesentery has been separated from the intestine at the time of evisceration.

In the following days (5-dpe) there was a significant growth in the size of the thickening that will form the intestinal rudiment (Figure 1D). This rudiment acquired an elongated oval or tear-shaped morphology, although in some cases or sections the growth could be rather irregular, where different sections of the same animal at different levels might show somewhat dissimilar morphologies. As early as this stage a small protrusion was also observed at the most distal tip of the growing rudiment. This outcropping eventually formed a smaller structure or appendix that was separated from the main mesenterial thickening by a constriction (see Figure 1E).

At 7-dpe the intestinal rudiment continued to increase in size and now had an area about 20 times larger than the 3-dpe rudiment (Figure 1E). At this stage the structure acquired a more rod-like structure, which can be seen in cross-sections as circular in shape. This rudiment sometimes showed numerous and deep folds formed by the coelomic epithelium. The appendix that formed at the tip was also evident (Figure 1E).

In the 5-7- dpe animals, three distinct regions were easily distinguished in cross-sectioned profiles of the gut rudiment. The first was a long section of mesentery that extended from its attachment in the body wall to the intestinal rudiment. This mesentery, although undergoing some morphological changes (see below) maintained a similar morphology to that of the uneviscerated animal. The second compartment was the intestinal rudiment that formed at the tip of the mesentery. This rudiment was separated from the rest of the mesentery by a constriction that became more evident as the thickening increased in size. The third compartment was the appendix that had grown at the very tip of the mesentery. This was much smaller than the intestinal rudiment, and appeared to be mainly composed of coelomic epithelial cells.

In the 10-dpe animals, the rudiment continued to grow in size and acquired a cylindrical shape (Figure 1F). In some animals a lumen formed. The luminal area comprised about 1/5 of the total cross-section area of the intestinal rudiment. Thus, the rudiment connective tissue and mesothelium continued to increase in size beyond merely an increase due to the formation of the lumen which itself caused a widening of the rudiment.

In summary, the first 10 days of regeneration were characterized by the formation of a tubular structure at the free end of the mesentery. Once formed this structure Meso-1 at about day 5, showing a 25-fold increase in size between 3 and 10 days of regeneration (Figure 2).

### A novel monoclonal antibody labels the intestinal and mesenteric mesothelium

One of the strategies used to dissect out the formation of the intestinal rudiment is to focus on particular cell populations involved in the regeneration process. Here we used a novel monoclonal antibody (Meso-1) that labels both major cell types of the gut mesothelium: the peritoneocytes, or coelomic epithelial cells, and the myocytes, or muscle cells, and follow the changes in this cell population during the first 10 days of regeneration.

**Figure 3:**
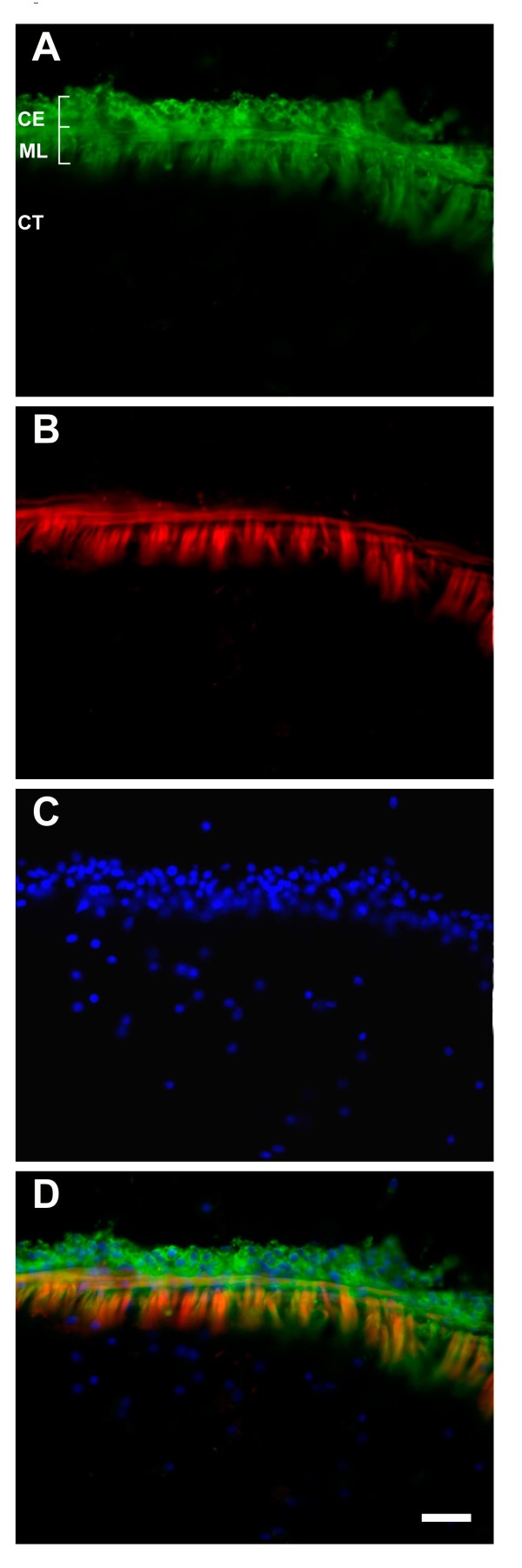
**Longitudinal sections of normal uneviscerated large intestine, showing Meso-1 immunoreactivity**. (A) Meso-1 labels the cells of the coelomic epithelium (CE) and the muscle layer (ML) (green). Cells of the connective tissue (CT) are not labeled. (B) The same section stained with rhodamine-labelled phalloidin only labels the muscle layer. (C) DAPI-labeled nuclei. (D) The colored overlay of the triple labeled section clearly shows that the Meso-1 antibody labels both mesothelium components while the phalloidin labeling is restricted to the muscle tissue. Bar = 25 μm

In the normal non-eviscerated animals, the antibody labeled the mesothelium of the intestine and mesenteries (Figure 3A). The label appeared to be distributed homogenously in the cytoplasm and, in the myocytes, it was particularly strong around the muscle contractile apparatus, but did not label the muscle filaments themselves (Figure 3B-D).

### Mesothelial cells ingress during intestinal rudiment formation

#### Rudiment

**Figure 4:**
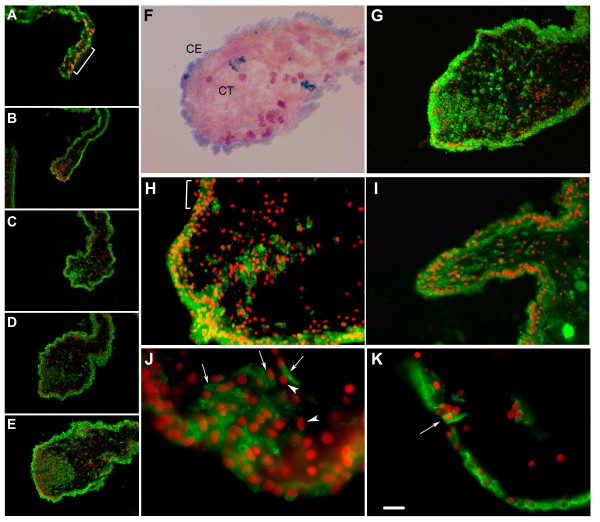
**Meso-1 labeling of the regenerating intestine (Days 1-5 of regeneration)**. Meso-1 labeling (green) and nuclei DAPI stain (red) show the process of ingression and the concomitant changes in the rudiment during regeneration. (A-E) The mass of cells at the distal tip of the rudiment is not observed at 1-dpe (A), becomes noticeable at 3-dpe (B) and increases in the subsequent days (C-E). Transverse sections of (F) Toluidene blue staining of 5-dpe intestine, shows the thickening of the mesenterial tip that forms the intestinal rudiment and differential staining of the coelomic epithelium (CE) and connective tissue (CT) compartments. (G) A similar section labeled with Meso-1 antibody highlights the ingressing cells. (H-I) Two additional examples of ingressing cells at 5-dpe, one is an invagination of the coelomic epithelium (H) while in the other, cells can be observed moving from the rudiment tip into the connective tissue (I). Figure H shows regions in the coelomic epithelia (bracket) that are thicker when compared to those in the 1-dpe mesentery (see bracket in Figure 4A). At higher magnification (J) some Meso-1 labeled ingressing cells show an elongated nuclei and cellular morphology (arrows) while other cells within the connective tissue are not labeled (arrowhead). (K) At the lateral side of the rudiment, an isolated ingressing cell (arrow) can be observed. Bar = (A) 70 μ m (B-E) 100 μ m (F-G) 65 μ m, (H-I) 30 μ m (J) 13 μ m (K) 20 μ m. All sections are from 5dpe animals except A (1dpe) and B (3-dpe).

During the growth of the intestinal rudiment, Meso-1 labeling showed that cells within the mesothelium also underwent a transition, particularly those in the area at or close to the injury site (Figure 4A-E). First, in the early stages of regeneration (1-3-dpe) the cells within the rudiment became cuboidal or rectangular and formed what appeared to be a single layer of coelomic epithelium (Figure 4A &4B). Second, the muscle layer disappeared from the growing found along the mesentery up to its tip (Figure 4A). More importantly, at 3-dpe, some of the epithelial cells began to ingress into the connective tissue at the tip of the mesentery (Figure 4B). The ingression was evident in animals at 5-dpe (Figure 4C-E). Labeling with Meso-1 showed that the ingressing cells retained the mesothelial labeling as they transitioned from the epithelial to a mesenchymal morphology. The ingression process continued during the next few days and appeared to peak at 5-dpe (Figure 4F-G). It is important to note that at this stage (5-dpe) some areas in the rudiment epithelium appeared to be several cells deep (see Figure 1D and 4H), contrasting from the usual one-cell coelomic epithelium found in normal mesentery (see Figure 1B and Figure 4A).

The ingressing cells formed a mass of cells adjacent to the tip of the mesenterial thickening. However, some labeled cells were also observed at a distance of up to 300 μ m from this cell cluster. The ingression site was usually associated with an invagination of the coelomic epithelium from which cells migrated into the surrounding connective tissue (Figure 4H &4J). However, not all ingression sites were equal (Figure 4I) and sometimes the ingressing cells were clearly observed to be entering directly from the overlying epithelium (Figure 4K). Many of the ingressing cells were elongated and appeared smaller than those of the coelomic epithelium. Their nuclei were somewhat different in shape; while most cells in the connective tissue or coelomic epithelia had round distinctive nuclei, some cells at the tip had an oval nuclei. Initially, this ingression appeared to take place only at the free margin of the mesentery adjacent to the area where the appendix-like structure had formed. However, at later stages, the ingression of cells that began at the mesenterial tip was observed to take place within the lateral areas of the rudiment and even within the adjacent mesentery (although not at the large numbers observed at the tip) (Figure 4K).

**Figure 5:**
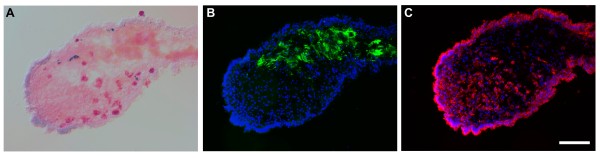
**Meso-1 and collagen immunoreactivity during intestinal regeneration**. Longitudinal sections of the 5 day regenerating intestinal rudiment showing (A) Toluidene blue staining (B) collagen immunoreactivity (green) and (C) Meso-1 labeling (red). Nuclei in B & C are stained with DAPI (blue). A and B are the same section while C is a similar section from the same animal. The figures clearly show that the area where the Meso-1 labeled cells are ingressing is devoid of collagen labeling, suggesting that the ingressing cells play a role in the remodeling of the extracellular matrix. Bar= 65 μ m

Labeling with anti-collagen showed that the area where the ingressing cells (Figure 5A-B) were present was the area devoid of collagen (Figure 5A&5C), suggesting that the ingressing cells were associated with the previously documented process of ECM remodeling [9] or that the accumulating mesenchymal cells created a collagen-free region. Thus, the intestinal rudiment in the 5-dpe animals was divided into three distinct areas: the distal margin of the mesentery, where the injury occurred, containing a mass of ingressing cells, the middle area with a smaller number of mesenchymal cells and no collagen bundles, and, next to the mesentery, an area with fewer cells but with some remaining collagen fibers. There was a very clear boundary between the area occupied by collagen fibers and the area devoid of them, and cells apparently involved in phagocytosis of ECM components could be observed (not shown).

**Figure 6:**
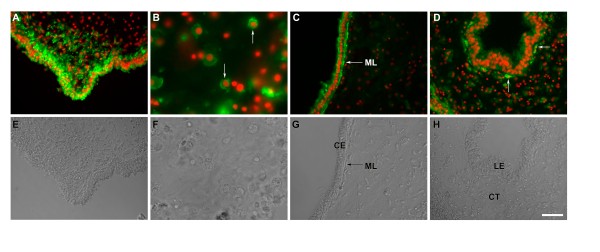
**Meso-1 labeling of the regenerating intestine (Days 7-10 of regeneration)**. Meso-1 immunoreactivity (A-D) and phase microscopy (E-H) of 7 and 10 day regenerating intestine. In the 7-dpe Meso-1 labeling (A&E) shows ingressing cells at the distal tip of the growing rudiment and (B&F) some immunoreactive cells within the connective tissue (arrows). In the 10-dpe rudiment, as regeneration progresses, Meso-1 labels (C&G) the forming muscle layer (ML) and (D&H) cells that underlie the basal lamina of the luminal epithelium (LE). CE-coelomic epithelium, CT-connective tissue. Bar = All 50 μ m except B& F 20 μ m.

At 7-dpe, the number of ingressing cells seemed to have diminished but could still be observed at the distal end of the rudiment (Figure 6A). Immunolabeled cells were observed in many areas of the connective tissue within the mesenterial thickening (Figure 6B). These cells were rather large and showed a prominent nucleus, abundant cytoplasm and some small extensions. The number of these cells was greater close to the ingressing cells at the tip, suggesting that these ingressing cells were giving rise to the large cells that could be found within the intestinal rudiment connective tissue.

At this stage a single layer of myoepithelial cells could be found within the coelomic epithelia (not shown). These cells originated from the overlying coelomic epithelia and were initially oriented in a circular manner but later provided the precursors of what will become the intestinal muscle layers [23].

At 10-dpe, the mesothelium of the rudiment remained strongly immunoreactive to Meso-1 and the forming muscle layer could be clearly observed (Figure 6C). At this stage, although some ingressing cells could still be observed, their number had greatly diminished. However, some of the immunolabeled large cells within the connective tissue appeared to be migrating toward the forming lumen and in some cases could be seen lying close to the basal end of the luminal cells (Figure 6D).

**Figure 7:**
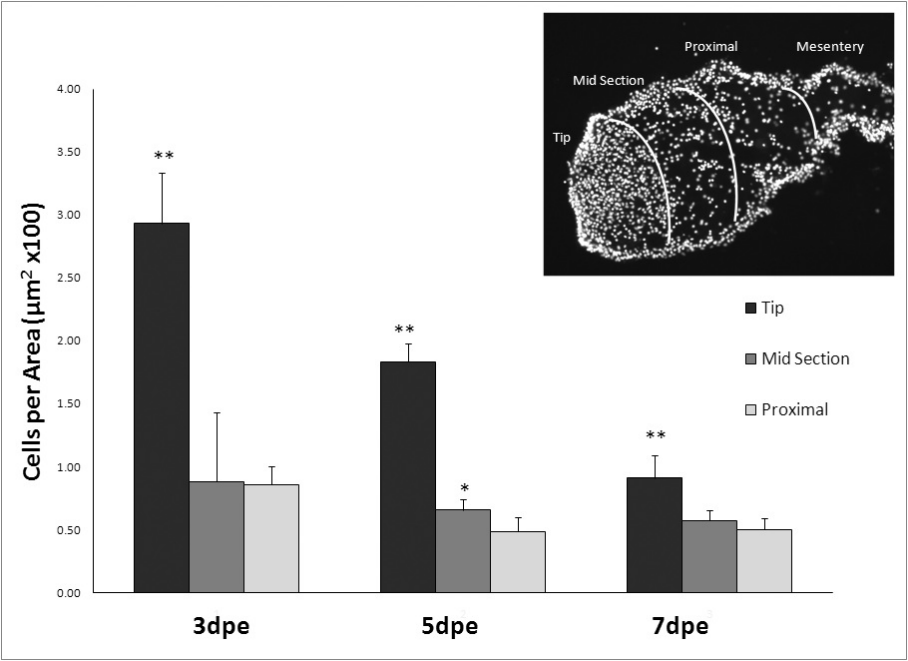
**Quantification of cell density in the regenerating rudiment**. The number of DAPI stained nuclei per μ m^2 ^was measured in tissue sections of regenerating rudiments of animals at 3-, 5-, and 7-days post evisceration. The connective tissue of the rudiment was subdivided into 3 different parts (tip, midsection and proximal). Insert shows an example of a rudiment with the areas that were measured. Each point represents the mean ± S.E. of at least three animals. Statistical analyses were done by comparing the density of cells in the proximal section to those on other areas of the rudiment at the same stage. Different from proximal *p < 0.05, **p < 0.01.

To quantify the ingression process we counted the number of cells within three areas of the rudiment connective tissue. These areas were: the tip (distal) area where the ingressing cell mass was found, the mid-section area where some Meso-1 labeled cells could be seen, and the proximal area to the mesentery where few if any Meso-1 labeled cells were found in the connective tissue (Figure 7). Results showed that at 3-, 5-, and 7-dpe the cell density within the mass of ingressing cells was much higher than in other areas. Moreover, in the 5-dpe stage there was also a larger density of cells in the mid section when compared to the area proximal to the mesentery suggesting that cells were indeed moving from the ingressing mass into the rudiment's connective tissue.

#### Mesentery

**Figure 8:**
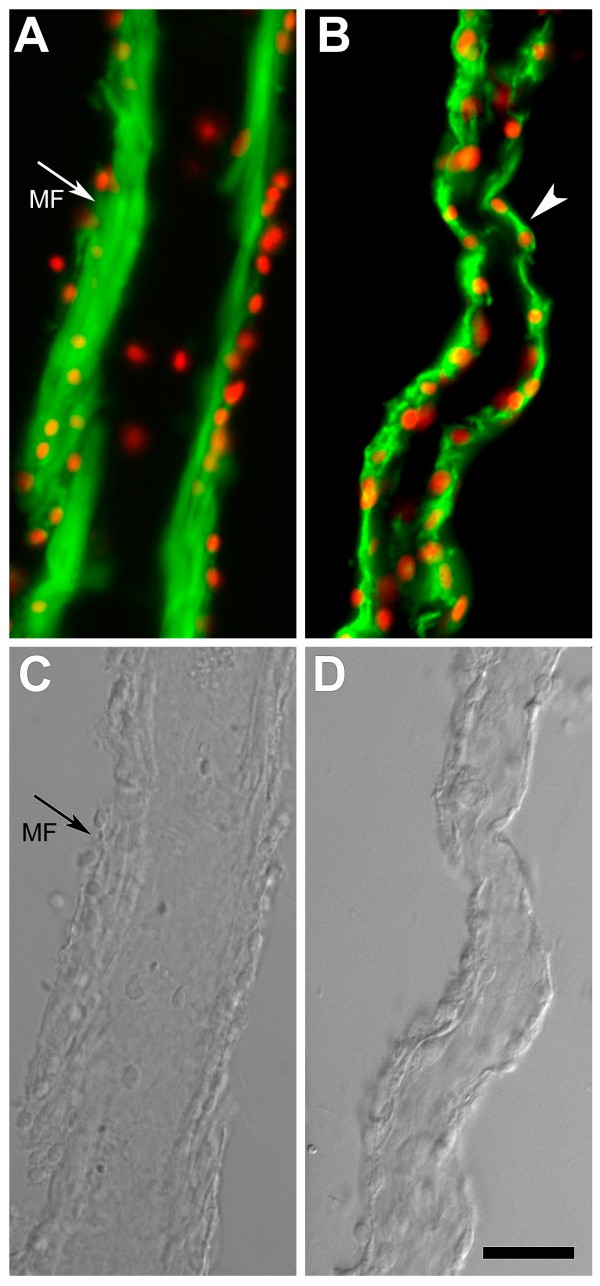
**Mesothelial changes in the mesentery during intestinal regeneration**. (A-C) Meso-1 labeling of the mesentery of normal uneviscerated animals shows a well-organized muscle layer with muscle fibers (MF) cut longitudinally and a weakly labeled overlying coelomic epithelium. (B&D) This organization is lost in the mesentery of 7-day regenerating animals where muscle fibers have disappeared and the mesothelium is mainly a one-cell layer (see cell labeled with arrowhead). Bar= 25 μ m.

Meso-1 also revealed striking changes in the mesothelium of the mesentery that did not become part of the intestinal rudiment. These changes were characterized by a disorganization of the muscle and overlying coelomic epithelium (Figure 8). During regeneration, the muscle fibers present in the normal mesentery (Figure 8A&8C) disappeared and the mesothelium became almost a single layer of coelomic epithelial cells (Figure 8B&8D). In addition, some cells from the mesothelium appeared to move toward the connective tissue layer in a manner reminiscent of what was observed in the growing rudiment (not shown). However, these cells moved as individual cells and not as a cluster as observed in the rudiment tip. The changes in tissue organization and cellular morphology observed in the mesentery were more pronounced the closer one got to the growing rudiment and to a lesser extent (if at all) close to the body wall. They also followed a temporal gradient, where the disorganization and disappearance of muscle was observed close to the rudiment at 3-dpe, but were not observed in other areas of the mesentery until 5- or 7-dpe, always in a gradient where changes were more pronounced the closer one moved to the rudiment and less obvious the closer one moved toward the body wall. Minimal (if any) changes were observed in the area of the mesentery attached to the body wall, where muscle fibers remained clearly visible and little disorganization was observed up to 10-dpe.

In summary, we have observed that following evisceration, mesothelial cells close to the tip of the mesentery undergo a dramatic change, from an epithelium/myocyte layer to a coelomic epithelium rather different from the peritoneocyte epithelium found in normal non-regenerating mesentery. More importantly, some of the coelomic epithelial cells at the tip of the mesentery ingress into the connective tissue. Ingressing cells acquire a mesenchymal phenotype and are mainly present in the area of the rudiment that is devoid of collagen. The ingression process begins at 3-dpe, peaks at 5-dpe, continues at 7-dpe and decreases at 10-dpe.

### Cell proliferation is minimal during early stages of regeneration

**Figure 9:**
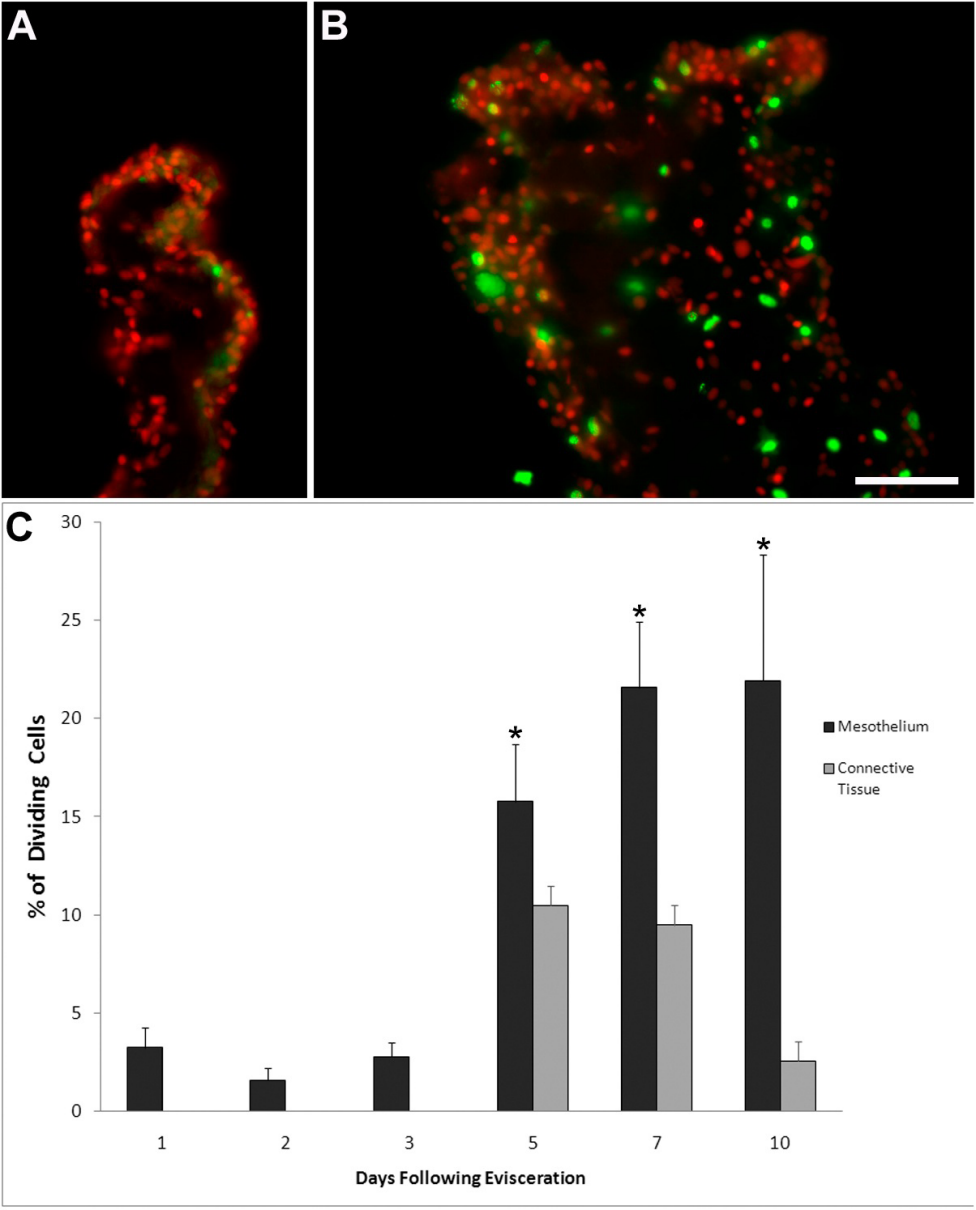
**Patterns of cell proliferation in regenerating intestinal rudiments**. Sections were labeled with an antibody against BrdU (green) and DAPI (red) to determine cell proliferation in the intestinal rudiment at (A) 2-dpe and (B) 5-dpe. Actively dividing cells were mainly observed in the coelomic epithelia of the rudiment of the 5-dpe animal with only one cell being labeled in the rudiment of the 2-dpe specimen. Bar= 50 μ m. (C) The percentage of BrdU-labeled cells or proliferation index was measured in the mesothelium (black) and connective tissue (gray) compartments of the regenerating intestinal rudiment. Each point represents the mean ± S.E. of at least three animals. *p < 0.05, **p < 0.01.

Previous studies from our laboratory showed low levels of cell division in the regenerating structure at 4-dpe; the earliest stage then studied [7]. To determine the contribution of cell proliferation to the thickening of the mesenterial tip and the formation of the intestinal rudiment, we studied the incorporation of BrdU in the S-phase of the mitotic cycle during the first 10 days of regeneration (Figure 9). A summary of the cell proliferation events in the regenerating rudiment can be observed in Figure 9C.

#### Rudiment

BrdU labeling showed low levels of proliferation at 1- and 2-dpe, (3.3 ± 1.0% and 1.6 ± 0.6% respectively) (Figure 9A). At 3-dpe, even though the thickening of the mesentery had begun, the percentage of dividing cells remained low and only 2.8 ± 0.7% of the cells incorporated BrdU.

At 5-dpe, the number of BrdU labeled cells increased in all areas of the intestinal rudiment, correlating with an increase in its size (Figure 9B). In the connective tissue area, 10.5 ± 2.7% of the cells now showed BrdU staining while in the epithelium 15.8 ± 2.9% of the cells showed BrdU incorporation. Although labeled cells were found throughout the rudiment coelomic epithelium, their distribution was somewhat heterogeneous with more labeled cells found in the distal mesenterial area (the area of the regenerating rudiment at the opposite end of the mesentery which corresponds to the tip of the regenerating rudiment). However, there were no particular differences in cell proliferation either on the protrusion at the tip or among those that were ingressing.

At 7-dpe, cell division continued to increase in the coelomic epithelium (21.6 ± 3.4% of cells in this layer incorporated BrdU). Dividing cells were now more evenly distributed along all areas of the regenerating rudiment. In contrast, in the connective tissue layer, cell proliferation remained at levels similar to those of 5-dpe rudiments with 9.5 ± 2.6% of the cells incorporating BrdU.

At 10-dpe, cellular proliferation in the coelomic epithelium remained high with 21.9 ± 6.4% of the cells incorporating BrdU. In contrast, cell division in the connective tissue decreased to 2.9 ± 0.3%. At this stage, the luminal epithelium was present in the intestinal rudiment of only one of three specimens used for the BrdU experiments. The luminal epithelium of this specimen showed high cell proliferation with 59.8% of the cells incorporating BrdU.

#### Mesentery

Cellular proliferation patterns were also studied in two regions of the mesentery: the area adjacent to the regenerating rudiment and the medial mesentery localized half way between the free edge of the mesentery and its attachment to the body wall. Cell division was observed in the mesothelium and the underlying connective tissue.

In general terms the pattern of cell division resembles somewhat that of the intestinal rudiment. At 1- to 3-dpe, around 1% of the mesothelial cells both in the mesentery adjacent to the rudiment or the medial mesentery showed BrdU staining. None of the cells in the connective tissue incorporated BrdU. A sudden change in the pattern of cell division was observed at 5-dpe. Cell division in the mesothelial layer of the mesentery increased dramatically in both the adjacent and medial areas. In the medial area, 8.1 ± 1.5% of the mesothelial cells showed BrdU labeling while a much higher percentage of cells were labeled in the adjacent area (19.6 ± 3.4%). In addition, cell division was first observed in the connective tissue layer of the mesentery, being higher in the medial area (31.0 ± 4.6%) than in the area adjacent to the rudiment (15.2 ± 3.1%).

At 7-dpe, the percentage of mesothelial cells labeled with BrdU in the area adjacent to the rudiment remained high (19.7 ± 4.5%). However, the percentage of dividing mesothelial cell in the medial segment had decreased to 2.8 ± 1.4%. In contrast, in the connective tissue layer, there were more labeled cells in the medial segment (18.5 ± 2.6%) than in the segment adjacent to the regenerating rudiment (7.6 ± 2.9%).

At 10-dpe, proliferation has decreased in the mesothelium of both adjacent (1.3 ± 0.9%) and medial (1.5 ± 1.1%) segments as well as in the connective tissue of adjacent (1.5 ± 1.4%) and medial (1.0 ± 0.8%) segments.

In summary, we have shown low levels of cell division in the regenerating intestine during the first 3 days of regeneration. Cell division increases at 5-dpe and is maintained up to 10 days in the mesothelium of the growing rudiment. The rate of cell division is always higher in the mesothelium than in the connective tissue layer.

### Mesenterial muscle de-differentiation begins during early stages of intestinal regeneration

The low levels of cell division observed during the first few days of intestinal regeneration suggested that, at least initially, cells forming the regenerating structure were not originating from dividing precursors. What then is the origin of the cells that form the mesenterial thickening? Previous work from our laboratory had shown that regeneration was associated with dramatic changes in the remaining mesentery, particularly with dedifferentiation of the mesenterial muscle [9,10]. Thus, we studied, the timing and the relative number of de-differentiating muscle cells in relation to the formation of the intestinal rudiment.

**Figure 10:**
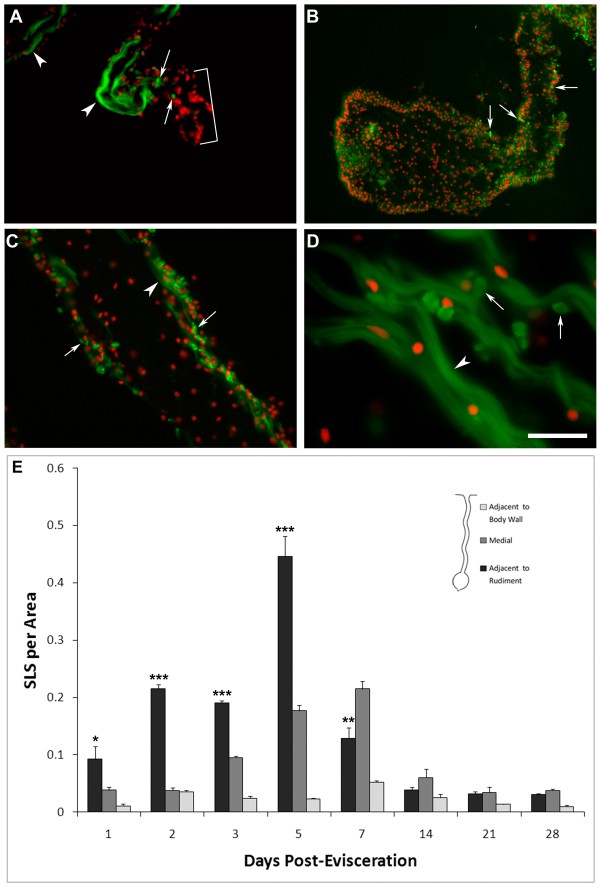
**Formation of spindle-like structures (SLS) by muscle cells during intestinal regeneration**. Double labeling of muscle fibers and SLS with rhodamine-labeled phalloidin (green) and cell nuclei with DAPI (red). (A) At 1-dpe, muscle fibers have disappeared from the tip of the mesentery (brackets) and SLS (arrows) are found close to the remaining muscle fibers (arrowheads). (B) At 5-dpe, the intestinal rudiment and adjacent mesentery are devoid of muscle fibers but some SLS (arrows) are present. (C) At 7-dpe, the area of the mid mesentery has SLS (arrows) and a few remaining muscle fibers (arrowheads), while (D) the mesentery close to the body wall has abundant muscle fibers (arrowheads) and only a few SLS (arrows). Bar = (A&C) 65 μ m, (B) 125 μ m, (D) 25 μ m. (E) The number of SLS was measured in different areas (corresponding to ~40,000 um^2^) of the intestinal rudiment and mesentery in regenerating animals from 1 to 28-dpe. In the mesentery adjacent to the rudiment, SLS show an increase from 1-dpe, peaking at 5-dpe. Areas of the mesentery distant to the regenerating structure (medial and distal) show a smaller amount of SLS and a peak at later stages. Each point represents the mean ± S.E. of at least three animals. ANOVA analysis showed significant differences in the mesentery adjacent to the rudiment. Asterisk show the results of t-test comparisons of different stages to dpe-28 *p < 0.05. **p < 0.01. ***p < 0.001.

The process of muscle dedifferentiation is characterized by condensation of filaments into spindle-like structures (SLSs), which are then often eliminated into the extracellular space [24]. Although this phenomenon has already been documented in the mesentery during regeneration [10], the temporal and spatial profile of SLS formation has not been documented. Using rhodamine-labeled phalloidin to detect the SLSs, we found that muscle dedifferentiation began at the free end of the mesentery as early as 24 hrs following evisceration (Figure 10A). Concomitant with the appearance of SLSs an increased disorganization and eventual disappearance of the muscle fibers was documented. Therefore, by the time the mesentery began to thicken and form the intestinal rudiment (3-dpe), there were few if any SLSs or muscle fibers within the mesothelium next to the mesenterial tip. The level of dedifferentiation increased in the following days and peaked at 5-dpe within the area of the mesentery adjacent to the intestinal primordia (Figure 10B&10E). At this stage no muscle fibers were observed within this section of the mesentery. In the following stages, particularly at 7- and 10-dpe, as the number of SLS began to decrease, new muscle fibers were observed.

Similar dedifferentiation processes were observed in other sections of the mesentery but fewer in number and at later stages (Figure 10C-E). In the mesentery adjacent to the intestinal rudiment, SLS formation started increasing at 24 hrs following evisceration and a peak was observed at 5-dpe, with a gradual decrease in the number of SLS thereafter. In the medial mesentery SLS formation began to increase (Figure 10C) and peaked slightly later. Similarly, disorganized muscle fibers and SLSs were also found in the section of the mesentery adjacent to the body wall (Figure 10D), but the number of SLS was much smaller and there was never a complete disappearance of the muscle fibers. SLS quantification showed that, in the mesentery adjacent to the rudiment, a peak of 150 SLSs per field of view was observed at 5 dpe, the peak in the medial mesentery was 70 (at 5-7 dpe) and in the mesentery close to the body wall it was 30 (at 7-dpe) (Figure 10E).

In summary, we have shown that muscle dedifferentiation begins very early during regeneration and occurs in a temporal and spatial gradient beginning at the free end of the mesentery soon after evisceration and moving toward the body wall in subsequent days.

## Discussion

We have now studied various cellular events that take place during the initial stages of intestinal regeneration in *H. glaberrima*. The results show that formation of the early intestinal regenerate occurs by the thickening of the mesenterial tip and that the initial steps in this process occur with little contribution from cell proliferation. On the other hand, concurrent with this process there are significant increases in muscle dedifferentiation adjacent to the regenerating structure. Finally, a previously undescribed mechanism by which coelomic epithelial cells ingress to form the mesenchyme at the tip of the regenerating structure is shown. Here we discuss the intestinal regenerative process in relation to what is known of regenerative processes in other echinoderms.

Our findings can be integrated into a working model of the cellular processes that form the intestinal rudiment. The initial event, is the healing of the wound by re-epithelialization. Contemporaneous with wound healing, the two cellular phenotypes within the mesothelium, myocytes and peritoneocytes, in the mesentery adjacent to the wounded edge, begin a process of dedifferentiation. The process is clearly observed in the myocytes due to the formation and elimination of SLSs, however, in other holothurian species, there is evidence that peritoneocytes also dedifferentiate, as determined by the loss of intermediate filaments [25]. Some of the dedifferentiated cells remain within the mesothelium and will give rise to the coelomic epithelium that surrounds the intestinal rudiment. As time proceeds, dedifferentiation continues in a retrograde gradient from the tip of the regenerating mesentery towards the body wall. The growing number of dedifferentiated cells provides the source for the coelomic epithelium to move as a sheet toward the tip of the mesentery. At about 3 days of regeneration, the cells of the coelomic epithelium begin to ingress into the underlying connective tissue layer, transforming from epithelial to mesenchymal phenotype. This ingression increases in the following days forming a mass of cells at the tip of the mesentery. As cells ingress and disseminate within the enlarging rudiment, it begins to acquire the tear-shaped morphology that shows an enlargement close to the tip. Ingressing cells undertake the changes needed to prepare the growth of the new structure. Among these changes are the remodeling of the ECM [9] and the overall growth of the structure necessary for the migration of luminal cells [7] and the formation of the intestinal lumen.

### Cell dedifferentiation and proliferation

The two cellular mechanisms that provide most of the cells for the regenerating intestinal rudiment, cell dedifferentiation and proliferation, appear to be shared by all regenerative events. They have been documented in regenerative processes not only in echinoderms but also in most animal groups.

#### Cell dedifferentiation

Cell dedifferentiation has been well described in echinoderms, where it has mostly been studied in muscle [12]. The regenerative processes where myocyte dedifferentiation is thought to be involved include muscle regeneration [24,26], cuvier tubule regeneration [27], and limb regeneration in crinoids [28,29] and asteroids [30]. In vertebrates, cell de-differentiation was first described over 50 years ago [31] and recent experiments have confirmed that it is indeed an important process for regenerating structures. Dedifferentiation has been mainly studied in amphibians and fishes where it has been documented in various cell types including iris cells [32], dermal fibroblasts [33] and muscle cells [34]. Moreover, recent tracing technologies have shown that dedifferentiated cells are incorporated into the regenerated structure, although their differentiation potential might be more restricted than previously thought [35-37].

In the holothurian, though we can document extensive cell dedifferentiation (by the presence of the SLS) we cannot certify as to the final destiny of these cells. These cells retain the Meso-1 label and some of them are probably incorporated into the coelomic epithelium. (It is important to remember that the echinoderm muscle cells are part of the mesothelium and thus lie over the same basal lamina as the epithelial cells). Recent experiments from our laboratory also suggest that dedifferentiating cells are not undergoing apoptosis. Although we have shown that during the first week of regeneration up to 5% of the mesothelial cells undergo apoptosis [21], most of the dying cells are observed in the area of the rudiment close to the mesentery, with very few apoptotic cells found within the mesentery. That the area covered by dedifferentiating cells is much more extensive than that where apoptosis is taking place suggest that there is no direct correlation between both processes. Therefore, although we propose that dedifferentiated muscle cells are actively participating in the regeneration process by becoming coelomic epithelial cells, at present, the technological limitations of our model system do not allow an in vivo study where the transformation and migration of live cells can be followed in real time to clearly determine the fate of the dedifferentiated cells.

#### Cell division

Cell proliferation has been a hallmark of the undifferentiated cells within the regenerating vertebrate blastema [4,31,38]. These cells, mainly found within the connective tissue underlying the epidermis proliferate, increasing their numbers, and eventually differentiate into the cells of the regenerated tissues. In contrast, in the holothurian intestinal system, cell proliferation takes place primarily in the coelomic epithelium. It begins slowly during the first week of regeneration, peaks during the second week of regeneration, and continues at lower levels for the following weeks [7]. We propose that cell division serves two purposes; first, it provides additional cells in the coelomic epithelium to counterbalance those that ingress. Second, it provides the cells necessary for the increase in area of the coelomic epithelium as the regenerating intestine expands and grows in size.

In this respect it is important to note that the proliferating coelomic epithelium will give rise not only to the peritoneocytes of the new intestine but also to the myocytes of the underlying circular and longitudinal muscle layers [12,23]. Immunohistochemical and ultrastructural studies done in various sea cucumber species suggest that these muscle cells originate from the dividing cells in the epithelium and differentiate as they move basally toward the basal membrane. However, in contrast to the ingressing cells they do not cross over the basal lamina [12]. In addition, the coelomic epithelium probably gives rise to the neurons within the mesothelium, but this differentiation has not been well studied.

It is important to highlight here some of the recent molecular data obtained in our laboratory [21]. The expression of two genes associated with inducing proliferation or inhibiting apoptosis, *survivin *and *mortalin*, is higher on the distal side of the rudiment, next to the injury site. However, what is really important is that the enhanced expression of both *survivin *and *mortalin *in the rudiment is indicative of a molecular transition in the mesothelial cells of the rudiment that goes hand in hand with the de-differentiation shown previously and with the increase in cell proliferation. Thus, the two genes serve as markers of the morphological and molecular changes that the mesothelium undergoes during regeneration. Even more interesting is the fact that initial expression of the both genes takes place in cells at the distal free margin of the mesentery corresponding to the cells that will undergo the EMT.

### Epithelial to mesenchymal transition (EMT)

The main difference or peculiarity of the holothurian regenerative structure is the origin of the mesenchymal cells. The holothurians show a hitherto undescribed regeneration mechanism by which cells from the epithelial layer ingress into the connective tissue layer and become mesenchymal cells. This appears to occur primarily along the gut autotomy plane and where a constriction forming an appendix has developed. EMTs have been well documented in developing embryos of animal species [39]. In echinoderms, in particular, they are important in the formation of the mesenchymal cells during gastrulation. Moreover, in adult animals, EMT can play important roles in wound healing, and cancer progression [40].

This mechanism contrasts with other regenerative events, particularly arm regeneration in ophiuroids and asteroids, as well as limb or fin regeneration in vertebrates where no cellular migrations are observed between the overlying epithelium (epidermis) and the underlying tissues. Nonetheless, the origin of cells for the tissue or organ regenerate is highly variable among the various animal groups. For example, the planarian blastemas are formed by neoblasts that migrate to the injury site [2,3]. In fish, fin regeneration blastemas are formed by migrating proliferating mesenchymal cells [41,42] while amphibian limb blastemas are formed by dedifferentiating cells in the injured limb [4,5].

It is also important to consider what might be significant differences between many regeneration model systems, such as limb regeneration and visceral regeneration. During limb regeneration the blastema is formed under the epidermis. This epithelial layer provides structural support and protection against loss of fluids and attack by pathogens. Ingression of epidermal cells into the connective tissue underneath, to occur, would bring with them pathogens that might be present in the external milieu. Visceral regeneration, on the other hand, occurs within the coelomic cavity, a compartment that (in our model system 3 days following evisceration) should be pathogen-free. Thus, the ingression of cells from the overlying epithelium should carry no risk to the regenerating rudiment. It is interesting, in this respect that reports of body wall muscle regeneration [24] and Cuvier tubule regeneration in holothurians [27] are also associated with a migration of cells from the overlying epithelium into the underlying tissues, albeit, to a lesser extent than the ingression observed during intestinal regeneration, since in these cases the cells do not cross the basal lamina. Two other cases in the echinoderm regeneration literature hint at ingression of mesothelial cells. The first is the formation of the anterior gut in sea cucumbers of the family Dendrochirota where the mesothelial cells have been postulated to give rise to the luminal epithelium [25]. Second is the regeneration of the digestive system in the crinoid *Antedon mediterranea *[43]. In this species, cells from the coelomic epithelium also appear to enter the underlying connective tissue and give rise to mesenchymal cells. The authors have also proposed that the entering cells eventually trans-differentiate into luminal epithelial cells thus reversing from an EMT to a mesenchymal-epithelial transition.

EMT during vertebrate visceral regeneration has also been shown. Zebrafish can regenerate their hearts following removal of up to 20% of the ventricular myocardium [44]. In this model system, epicardial cells undergo EMT, invading the wound and generating endothelial and smooth muscle cells of the vasculature [45]. Thus, the available data suggests that in both vertebrates and invertebrates EMT events could be playing major roles in visceral regeneration.

### What is the role of the ingressing cells?

Although at present it is difficult to clearly establish the role of the ingressing cells in the sea cucumber, our understanding of the ongoing cellular events do provide some possible explanations. First, they are probably involved in the remodeling of the ECM that occurs in the regenerating structure. Ingressing cells, might give rise to the phagocytic amebocytes found 3-5 days after evisceration that are responsible for the degradation of the extracellular matrix, particularly of the collagen component [9,10]. Moreover, there is a temporal and spatial correlation where ECM remodeling occurs at the same time that cells are ingressing and the area in the intestinal rudiment where the ingressing cells are found is the area that is devoid of collagen. Ingressing cells might have other roles, among these the formation of the new ECM, being the precursor to new cells in the regenerating mesenchyme or even participating in the formation of the intestinal lumen. In the latter, it is important to highlight that some of the ingressing cells appear to contact the luminal cells in the 10dpe animals and that this interaction might be essential for the formation of the luminal basal lamina and the maintenance of the luminal epithelial layer.

On the other hand, it is important to emphasize that ingressing cells in the holothurian are not necessarily equivalent to the "blastemal cells" found in regenerating amphibian limbs [4] or fish fin blastemas [42,46] nor to planarian neoblasts [47]. The main difference, other than their origin, is shown by the limited proliferation activity; there is less BrdU incorporation in these cells and proliferative events last less than in cells of the overlying coelomic epithelium, or the forming luminal epithelium. Thus, once again it seems that the mesothelial cells are the key players in providing cells for the regenerative structure.

### Regeneration in echinoderms

Echinoderm regeneration studies have mainly focused on arm regeneration in crinoids [48], ophiuroids [49,50] and sea stars [30,51] and in muscle and visceral regeneration in holothurians [12,24,52]. However, instead of showing similar processes involved in the regeneration of different structures or organs, these studies have focused on the differences among the echinoderm groups. Take for example the studies by Candia Carnevalli's group that showed that the regenerating arm of the crinoid *A. mediterranea *is mainly formed by undifferentiated proliferating (BrdU-incorporation) mesenchymal cells underlying the epidermis [28,29]. In contrast, the cells that give rise to the regenerated arm in two sea star species; *Leptasterias hexactis *[51] and in *Asterias rubens *[30] appear to originate from dividing cells in tissues of the arm stump that migrate into the injury area.

The differences in regeneration processes among the echinoderms might be due to factors that have to do with availability of cell precursors, such as the number of cell precursors or the distance where they can be found or produced in relation to the injury. The echinoderm coelomic epithelium has been considered a tissue capable of giving origin to a large number of cell types, including myocytes, peritoneocytes, coelomocytes and possibly others [12,53]. Thus, during intestinal regeneration once the wound is healed (following the evisceration rupture) the mesentery tip that will give rise to the intestinal primordia is surrounded by cells capable of producing precursors to many cell phenotypes. It also needs to be taken into account that the number of cells available within the mesenterial connective tissue is very small and the nearest source of cells, other than the mesothelium, would be the body wall. In this case, cells would have to migrate a considerable distance via the mesentery toward the tip where intestinal regeneration takes place [54]. In this scenario, the cells of the mesothelium, are capable of providing most of the cells needed for intestinal regeneration.

Therefore, what we propose is that echinoderm regeneration relies on both cell dedifferentiation and proliferation; in cases where large number of cells are needed to regenerate a body structure both events can be observed. Otherwise, in some cases where the dedifferentiating cells are nearby or small number of cells are needed, one of the two mechanisms can take place preferentially.

## Conclusions

Our data show that three events are important in forming the intestinal rudiment during the process of intestinal regeneration. The initial event is the dedifferentiation of the mesenterial muscle layer that begins near the free-tip of the mesentery and spreads gradually toward the body wall. Second, is the ingression of cells at the tip of the mesentery providing some of the mesenchymal cells for the connective tissue. Third, is cellular proliferation. Cell division begins later during the regeneration process and mainly occurs within the mesothelium of the growing rudiment and mesentery. Two of these events, cell dedifferentiation and proliferation are common to many regenerative processes, both in vertebrates and in invertebrates. The observed epithelial to mesenchymal transition might be particular to the regeneration of visceral organs.

Our results provide a clearer view of the cellular events involved in the formation of the intestinal rudiment during visceral regeneration. They highlight the dedifferentiation, proliferation and epithelial-mesenchymal transition of mesothelial cells and their possible role as precursors of the new intestinal cells. Nonetheless, new questions emerge that need further investigation. Among these are the identification of the physical and molecular factors associated with the EMT and dedifferentiation, as well as the possible role of the ingressing cells in the formation of the intestinal lumen.

## Methods

### Animals

Adult individuals of *Holothuria glaberrima *were collected from the northern coast of Puerto Rico. They were kept in seawater aquaria. Evisceration was induced chemically with intracoelomic injections of KCl 35 mM (3-5 mls per animal). At least three animals were used for each experiment at stages 1-, 2-, 3-, 5-, 7-, and 10- days post-evisceration (dpe).

### Antibody production

Two mice were immunized with a cellular homogenate obtained by scraping the coelomic epithelium of 7-dpe regenerating longitudinal body wall muscles of *H. glaberrima *[55]. Fifty microliters of the emulsion (equal volumes of TiterMax (Sigma) and the extracted tissue solution) were injected intraperitoneally in each mouse. After thirty days, the serum was extracted and utilized as a polyclonal antibody source for immunohistochemistry.

One animal was boosted one week before spleen dissection and used for the production of monoclonal antibodies. The fusion was performed by the stirring method [8,56] with a spleen:myeloma (SP20) ratio of 6:1. The supernatant of wells exhibiting good hybridoma growth were used for immunohistochemical assays of holothuroid body wall. We selected the Meso-1 clone due to its labeling of the mesothelium of both the intestine and the body wall muscles.

### Immunohistochemistry

Animals were anesthetized by keeping them in 0.5% 1, 1, 1-Trichloro-2-methyl-2-propanol hydrate in seawater for 20-30 min. The regenerating digestive tube was dissected out and fixed overnight in 4% paraformaldehyde, in 0.1M PBS, rinsed with the same buffer three times for 15 min and cryoprotected in 30% sucrose/PBS until sectioned. The cryosections (20 μ m) were obtained using a Leica CM1850 cryostat.

Immunohistochemical techniques have been described previously [8,57]. In brief, the primary antibody was left overnight and sections were placed in a humid chamber at room temperature. The next day, the slides were washed three times with 0.1 M PBS for 15 min. Secondary antibody was applied for an hour. Following three more PBS washes, slides were mounted in buffered glycerol containing DAPI and observed and analyzed using a Nikon Eclipse E600 fluorescent microscope. The antibodies used were Meso-1 and anti-collagen HgCol [9]. Those slides that were stained with polyclonal primary antibodies, were treated with 1/50 goat serum prior to the application of the primary antibody to reduce non-specific background fluorescence.

In some cases, immunofluorescence was performed in fixed sections as described and after incubation with the secondary antibody, slides were incubated for one minute in toluidene blue. Slides were washed two additional times with PBS, 15 min each before mounting.

In other cases, muscle labeling was done using fluorescent-labeled phalloidin by adding it during the incubation with the secondary antibody. Phalloidin-FITC (Sigma P5282), or Phalloidin-TRITC (Sigma P1951) were used at final concentrations of 1:1,000 and 1:4,000 respectively.

Measurements of the rudiment area were done using ImageJ software (http://rsbweb.nih.gov/ij/). At least 3 sections were measured from each animal and at least 3 animals were used for each stage.

### Cell quantification

Tissue sections from regenerating animals at 3-, 5- and 7-dpe regeneration stages were immunolabeled with the Meso-1 antibody and DAPI. Using the Meso-1 labeling the area of the connective tissue of the rudiment was subdivided into 3 parts: the distal area where the ingressing cell mass was present and most, if not all, cells were Meso-1 labeled, the midsection area where large, individual Meso-1 labeled cells were found together with unlabeled cells, and the proximal area, adjacent to the mesentery where most of the cells were not labeled with Meso-1. The area of each sub-division was measured using ImageJ software (http://rsbweb.nih.gov/ij/) and the number of DAPI stained nuclei counted. The number of DAPI nuclei divided by the subdivision area was used to determine the cell density in each area. The cell density for each area was compared to other areas of the same stage using t-test.

### Cell division

Regenerating animals at various regeneration stages (1-, 2-, 3-, 5-, 7-, and 10-dpe), were injected with BrdU (SIGMA, Cat. #B5002) at a concentration of 0.5 mg/100 μL per kg (animal wet wt). Animals were kept in an aquarium and sacrificed 4 hrs after the injection.

The immunohistochemistry protocol described above was followed with some additional steps that include: A wash with Triton 100x (0.2%) for 15 min prior to the application of the primary anti-BrdU antibody. Two washes with 0.1M PBS for 15 min. A one-hour treatment with 0.05M HCl. Another PBS wash followed by treatment with the murine monoclonal anti-5-bromo-deoxyuridine (GE Healthcare Code: RPN 202). The antibody was diluted 1:4 in RIA Buffer prior to use. Slides were mounted as described above.

BrdU immunoreactive cells were counted and the number was normalized relative to the total number of cells labeled with DAPI within the visual field using the 40x objective. The ratio of BrdU/DAPI labeled cells was compared between the different regenerating stages. At least four animals were used per stage and at least four sections were analyzed per animal.

### SLS quantification

Myocyte dedifferentiation has been described in several echinoderm species [12,24]. A hallmark of muscle dedifferentiation is the formation of spindle-like structures (SLSs). These are cell-derived structures that contain portions of the contractile apparatus of the dedifferentiating cells. Thus, the number of SLSs found in a tissue correlate with the number of cells undergoing dedifferentiation. In order to determine the extent of muscle dedifferentiation in the regenerating intestine, the number of SLSs was quantified at each stage of regeneration.

SLSs were labeled using rhodamine-labelled phalloidin as described elsewhere [55]. To quantify the changes in SLS formation two methodologies were used. First, the number of SLS in a segment of mesentery measuring ~40,000 um^2 ^was counted. This was done by using the 20X objective and measuring the tissue within the microscope field of view. Alternatively, the number of SLS and the mesentery area where they were present was measured to establish the SLS density. Both techniques provided similar results in terms of the pattern of SLSs present in different parts of the mesentery. The number of SLSs/per area were measured at three different levels of the mesentery; near the body wall, medial and near the regenerating rudiment. All areas used for measuring SLSs were of similar size (~40,000 um^2^). At least three animals were used for each stage and at least two sections from each animal were counted.

### Statistical Analyses

Statistical analyses were done using t-test and ANOVA. All values are reported as mean ± standard error.

## Authors' contributions

JGA conceived the study, participated in the interpretation of the results and wrote the manuscript. GVT carried out most of the immunohistochemical results with Meso-1 and obtained and analyzed the toluidene blue data. JF performed and analyzed the BrdU experiments. RR carried out the experiments with collagen. ARC carried out the experiments and analyzed the data on SLSs. JESM produced the Meso-1 antibody. KT contributed to the interpretation of wound healing and rudiment formation. All authors read and approved the final manuscript.
